# Response of Tomato (*Solanum lycopersicum* L.) Genotypes to Heat Stress Using Morphological and Expression Study

**DOI:** 10.3390/plants11050615

**Published:** 2022-02-24

**Authors:** Abdulhakim A. Aldubai, Abdullah A. Alsadon, Hussein H. Migdadi, Salem S. Alghamdi, Sulieman A. Al-Faifi, Muhammad Afzal

**Affiliations:** 1 Department of Plant Production, College of Food and Agriculture Sciences, King Saud University, Riyadh 11451, Saudi Arabia; hakimaldobai@gmail.com (A.A.A.); alsadon@ksu.edu.sa (A.A.A.); salem@ksu.edu.sa (S.S.A.); salfaifi@ksu.edu.sa (S.A.A.-F.); mmushtaq@ksu.edu.sa (M.A.); 2 National Agricultural Research Center, Baqa, Amman 19381, Jordan

**Keywords:** tomato, heat shock protein, transcription factor, expression, qPCR

## Abstract

Due to unfavorable environmental conditions, heat stress is one of the significant production restrictions for the tomato (*Solanum lycopersicum* L.) crop. The tomato crop is considered an important vegetable crop globally and represents a model plant for fruit development research. The heat shock factor (HSF) gene family contains plant-specific transcription factors (TFs) that are highly conserved and play a key role in plant high-temperature stress responses. The current study was designed to determine the relative response of heat stress under three different temperatures in the field condition to determine its relative heat tolerance. Furthermore, the study also characterized heat shock genes in eight tomato genotypes under different temperature regimes. The expressions of each gene were quantified using qPCR. The descriptive statistics results suggested a high range of diversity among the studied variables growing under three different temperatures. The qPCR study revealed that the *SlyHSF* genes play an important role in plant heat tolerance pathways. The expression patterns of HSF genes in tomatoes have been described in various tissues were determined at high temperature stress. The genes, *SlyHSFs-1*, *SlyHSFs-2*, *SlyHSFs-8*, *SlyHSFs-9* recorded upregulation expression relative to *SlyHSFs-3*, *SlyHSFs-5*, *SlyHSFs-10*, and *SlyHSFs-11*. The genotypes, Strain B, Marmande VF, Pearson’s early, and Al-Qatif-365 recorded the tolerant tomato genotypes under high-temperature stress conditions relative to other genotypes. The heat map analysis also confirmed the upregulation and downregulation of heat shock factor genes among the tomato genotypes. These genotypes will be introduced in the breeding program to improve tomato responses to heat stress.

## 1. Introduction

The tomato (*Solanum lycopersicum* L., 2n = 24) is a self-pollinated crop that belongs to the Solanaceae family, ranking second to potato in the world’s vegetable production. In the Solanaceae family, there are more than 3000 different types of species [1], which includes the tomato, one of the world’s most significant vegetables [2]. According to FAOSTAT [3], the total cultivated area by tomatoes around the world was estimated at around 5.8 million hectares with a production of 233.5 million tons. In Saudi Arabia, the devoted area to tomato production was 13.3 thousand hectares with average productivity of 23.01 tons per hectare. Heat stress or heat shock is defined as a temperature increase of 10–15 °C above the optimal temperature [4]. Heat stress is a multidimensional function that is determined by the rate of temperature rise and the total length of exposure [5]. In specific climatic zones, the intensity of heat stress is primarily influenced by the frequency of heat shock and the length of high day/night temperatures. Thermotolerance refers to a plant’s ability to withstand exceptionally high or low temperatures while still producing a profit. According to reports, the tomato crop in some parts of the world is subjected to extreme temperatures regularly. Heat stress has a significant impact on tomato reproduction. High temperatures can cause the male gametophyte to abort, resulting in a decline in fruit set. By 2100, the predicted rise in air temperature (1.5–11 °C) will have a significant impact on crop productivity [6]. As a result, agricultural plant reproductive behavior in these harsh settings must be thoroughly investigated [7].

Tomato fruit production is influenced by high-temperature stress, despite its capacity to thrive in a variety of climates, because increases in day/night temperatures above 26/20 °C, respectively, can severely affect fruit setting and output [8]. With climate change, developing heat-tolerant cultivars that can withstand high temperatures and other abiotic pressures is a top goal. Commercial tomatoes, unlike *Solanum chilense*, have a limited heat tolerance capacity. Heat stress is the major hazard to agricultural output in many parts of the world because of global warming [9]. Increasing the daytime temperature above 25 °C reduces the number of fruits, the weight of the fruit, and the number of seeds per fruit [10]. Short-term exposure to extremely high temperatures (45 °C) can cause programmed cell death (PCD), cytochrome c release, and the induction of caspase-like enzymes [11]. Plants in the reproductive stage are more vulnerable to high temperatures relative to the vegetative stage [12]. Due to elevated summer temperatures, tomato production is halted in numerous countries [13]. The lack of heat tolerance in most tomato cultivars makes it difficult to cultivate them in areas where temperatures exceed 38 °C or more for part of the growing season, even if only for a short time [14]. High temperatures interfere with physiological and biochemical development, resulting in lower fruit yields [15]. Tomato plants are vulnerable to high temperatures that can stimulate the flower dropping [16] and reduce the fruit yield [17]. Temperature increases have a deleterious impact on the pollen grain, particularly during the initial stage, resulting in poor pollen germination and reduced pollen tube growth [18]. High temperatures not only diminish flowering and fruit set on the plant but also impair the development and maturity of the fruit, lowering crop production [19]. As a result, improving the heat tolerance of crops by molecular manipulation is critical. In plant cells, heat stress causes many heat-labile proteins to denature and increases damaging reactive oxygen species (ROS) [20].

When exposed to heat, heat-shock genes are quickly expressed, resulting in a rapid buildup of heat-shock proteins (HSPs). The heat shock transcription factors (HSFs) regulate HSP expression primarily at the transcriptional level, and they play an important role in high-temperature stress responses [21]. Plant HSF genes have been extracted from a variety of species since they were first discovered in tomatoes [22]. In an earlier study, there were many HSF genes identified in Arabidopsis (21) and rice (*Oryza sativa* L.) (25), respectively [23]. The HSF family, like many other TFs, has a modular structure with highly conserved domains [24]. The conserved structure includes the N-terminal DNA-binding domain (DBD) and the heptad hydrophobic repeats (HR-A/B) engaged with the nuclear localization signal (NLS) domain [23]. A C-terminal activation domain (CTAD) and a nuclear export signal (NES) domain are also found in several HSFs [25]. HSFs work by inducing a highly conserved heat shock element (HSE) in the promoters, which contains motifs in alternating orientations. Shim et al. [26] found that class A (HSFs) are engaged in transcriptional activation and environmental stress responses, whereas class B (HSFs) act as repressors of gene expression [27,28]. HSFB1 serves as a transcription co-activator with class A HSFs in Arabidopsis and as a repressor in tomatoes, according to previous studies [28]. Many species, including Arabidopsis, Chinese cabbage, rice, maize, Triticum aestivum, pepper, and grasses, have been extensively studied for the HSF gene family [29,30]. The current study was designed to explore the tomato genotypes collected from different hot climate regions of Saudi Arabia and to determine their response under field conditions and expression levels using different heat shock transcription factors (HSFs) of the *SlyHSFs* genes.

## 2. Results

The descriptive statistics results suggested that there were a wide range of diversity present among the studied characters, i.e., plant height (cm), thickness (cm), fresh weight leaf (FWL), fresh weight stem (FWS), number of branches (NOB), fresh weight/plant (FW/p), dry weight leaf (DWL), dry weight stem (DWS), total dry weight/plant (TDW/plant), and leaf area/plant (LA/p) under three different temperature regimes (high temperature, medium temperature, and low temperature) at vegetative stage (Table 1). The mean data for all genotypes under three growing seasons are presented in Supplementary Table S1.

**Figure 1:**
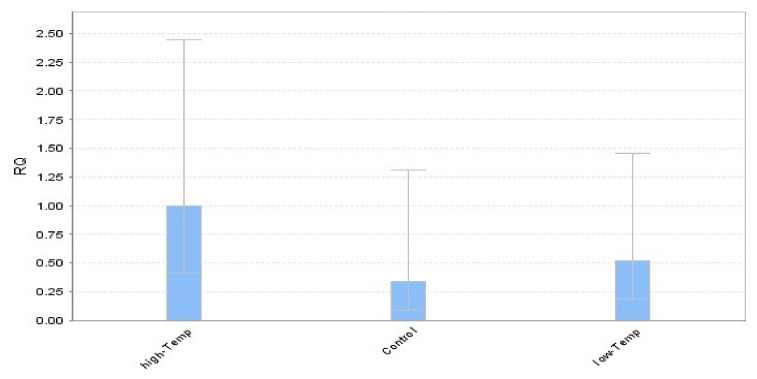
Relative quantification (RQ) of the heat shock transcription factor (*SlyHSF-01*) gene under different temperature regimes in tomato genotypes. (High temperature = 42 °C, low temperature = 12 °C, medium temperature = 25 °C). Beta-actin gene was used as an internal control for quantitative expression.

The study was conducted to determine the comparative expression profiling of the heat shock transcription factor (HSF) coding genes in the tomato cultivars. For that purpose, the tomato genotypes were exposed to different temperature regimes (high temp, medium temp, and low temp), and samples were collected for RNA extraction. The cDNA was synthesized for the comparative expression of the selected genotypes and actin was used as an internal control to normalize the reactions. For this purpose, 11 (HSF) genes were used to evaluate the expression (Table 2). As HSF genes participate in pathways associated with heat shock, a quantitative PCR analysis has been carried out in the tomato to comprehensively detect HSF gene expression functions. The relative expression was determined using the ∆∆Ct among each primer used during the study. Furthermore, the genotype evaluation was performed by generating the graph on the CT mean value of each primer. Primer 1 (*SlyHSF-01*) results are presented in Figure 1.

From the figure, it was predicted that all the genotypes recorded upregulation of the heat shock transcription factors (HSFs) at high temperatures as compared to medium and low temperatures (Figure 1). Furthermore, the expression was also recorded more at low-temperature collections of the samples as compared to the control (medium temperature).

**Figure 2:**
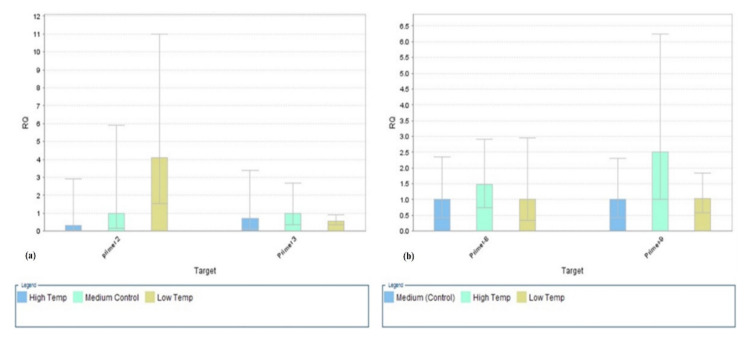
Relative quantification (RQ) of the heat shock transcription factor (*SlyHSF-02* and *3*, (**a**)) (*SlyHSF-08* and *09*, (**b**)) genes under different temperature regimes. (High temperature = 42 °C, low temperature = 12 °C, medium temperature = 25 °C). Beta-actin gene was used as an internal control for quantitative expression.

**Figure 3:**
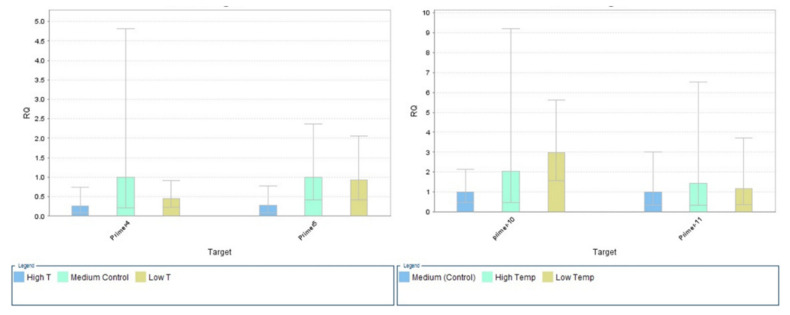
Relative expression of heat shock transcription factor genes under different temperature regimes left, *SlyHSF-04* and *5*; right, *SlyHSF-10* & *11* (high temperature = 42 °C, low temperature = 12 °C, medium temperature = 25 °C). Beta-actin gene was used as internal control for quantitative expression.

**Figure 4:**
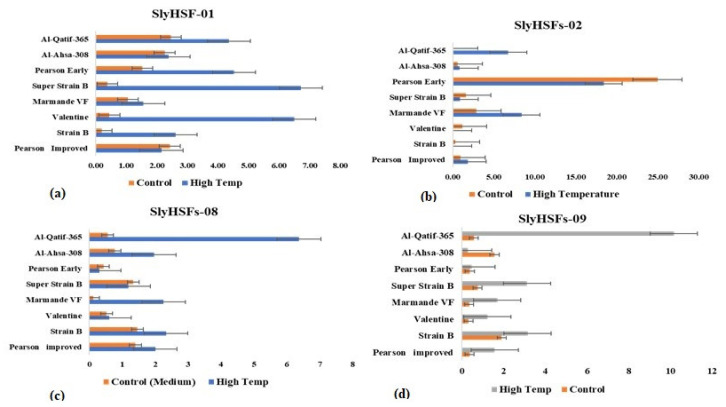
Fold change expression difference of individual tomato genotypes using different heat shock transcription factors genes ((**a**) *SlyHSF-1*, (**b**) *SlyHSF-2*, (**c**) *SlyHSFs-8*, (**d**) *SlyHSFs-9*).

The expression of the *SlyHSF-02* genes for all tomato genotypes was presented in Figure 2. The results were quite opposite as compared to the *SlyHSF-01* recorded down-regulation of the HSF function; however, some genotypes (Al-Qatif-365, Al-Ahsa-308, Marmande Vf, and Pearsons improved) recorded upregulation under high temperature. The maximum expression was recorded under lower-temperature treatment, while the minimum was under high temperature relative to the control (Figure 2). While at the same time, *SlyHSF-03* also recorded maximum expression in high-temperature treatment relative to control and low temperature (Figure 2). The *SlyHSF-04* and *SlyHSF-05* expression results are presented in Figure 3. The maximum expression was recorded under control (medium temp) regimes as compared to other temperature treatments. At high-temperature treatments, the HSP genes were down-regulated in both cases (*SlyHSF-04*; *SlyHSF-05*). The comparative expressions of the *SlyHSF-08* and *SlyHSF-09* are presented in Figure 4. The HSF genes were upregulated in the *SlyHSF-08* as well as *SlyHSF-09* primers relative to other temperature treatments.

The comparative expression of result for the primers, *SlyHSF-10* and *SlyHSF-11*, are presented in Figure 3. *SlyHSF-10* recorded upregulation of HSF and was significant as compared to other treatments. Similarly, the comparative expression of the *SlyHSF-11* also recorded upregulation of HSF genes while downregulation was recorded under control treatment (Figure 3).

**Figure 5:**
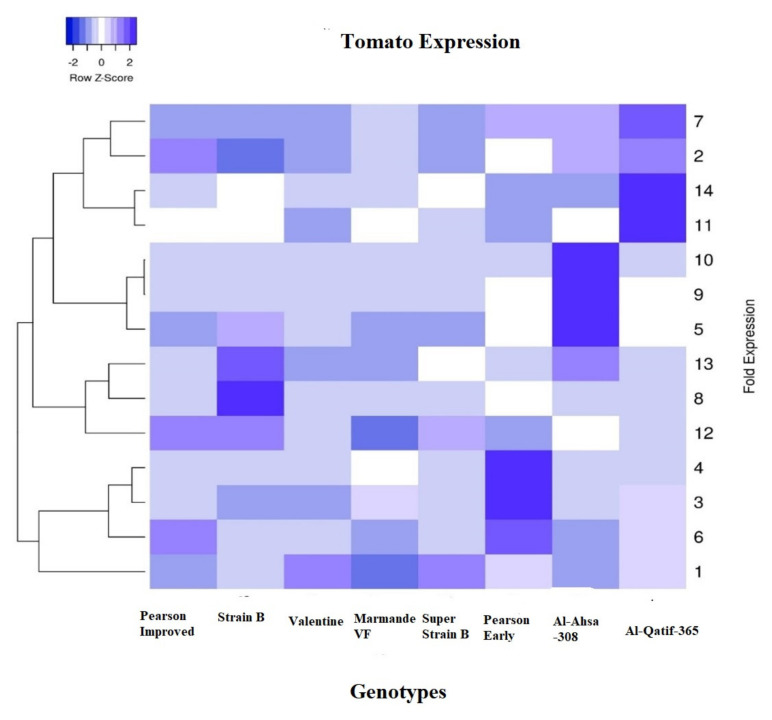
Heat map of heat shock transcription factor (HSF) samples collected at high-temperature stress and medium stress in tomato genotypes. The expression-based hierarchical clustering of genes is presented to show various gene clusters. The color intensity represents the degree of change in each tomato genotype; dark blue represents upregulation, and light blue represents downregulation.

The fold expression of each tomato genotype was also determined under the high temperature relative to the control (medium temperature). The gene (*SlyHSFs-01*) recorded significant fold change expression relative to the control (Figure 4a). The maximum expression was recorded in Super Strain B (7.0) followed by Valentine (6.5) under high-temperature stress relative to the control. Similarly, *SlyHSFs-2* also recorded significant upregulation under high-stress temperature in Marmande VF (8.38) and Al-Qatif-365 (Figure 4b). The upregulation was also recorded as significant in Pearson Early, but relative to the control, it was downregulated under high-temperature stress conditions. Similarly, Al-Qatif-365 recorded the highest expression when *SlyHSFs-8* (Figure 4c) and *SlyHSFs-9* (Figure 4d) genes were used, respectively, followed by strain B and Marmande VF tomato genotypes under high-temperature stress conditions. The heat map of the heat shock transcription factors (HSFs) shows that the expression-based hierarchical clustering of genes confirms the upregulation and downregulation of heat shock factor genes among the tomato genotypes (Figure 5).

## 3. Discussion

Characterizing heat stress tolerance genotypes is significant to figuring out the target genotypes’ responses under different temperature regimes. The current study was designed to determine the heat-resistant genotypes based on phenotypic data as well as gene expression data under different temperature regimes. The combination of temperatures, relative humidity, and solar radiation defined the growing conditions. In heat stress research, temperatures are frequently given more weightage than relative humidity [31]. High relative humidity (over 70%) can, on the other hand, exacerbate the effects of high temperatures on crops [32]. When testing for heat stress tolerance in tomatoes, more attention should be paid to both temperature and relative humidity [33]. The results suggested that the vegetative characters recorded a significant effect from heat stress, and it was also found that there were significant differences present between the genotypes and different temperature regimes. The plant height (cm), thickness (cm), fresh weight leaf (FWL), fresh weight stem (FWS), number of branches (NOB), fresh weight/plant (FW/p), dry weight leaf (DWL), dry weight stem (DWS), total dry weight/plant (TDW/plant), and leaf area/plant (LA/p) were recorded in the Valentine genotype, followed by the Pearson improved genotype; however, the minimum was recorded in the Al-Qatif tomato genotype. Similar results were reported by Ayenan et al. [33], suggesting that different heat stress periods affect the vegetative and yield components in tomato cultivars.

The heat shock transcription factor was found 30 years ago and takes part in the genes under thermal stress, which can produce thermal shock protein, i.e., it exhibits a high degree of gene expression during thermal stress with a transcriptive activation. When a plant is exposed to thermal stress, however, the expression of heat-shock genes rises, resulting in a rapid accumulation of heat-shock proteins (HSPs). When it was originally identified from tomatoes, the understanding of heat transcription factors had just begun [34]. It has demonstrated a remarkable effect in assisting plants, particularly *Solanaceous*, in withstanding heat stress that is approximately 5–10 °C over the plant’s usual growing temperature. Given that global warming is increasing marginal land and posing a threat to the production of vital food and cash crops, such as solanaceous, it is critical to investigate heat shock transcription factors that can help plants endure heat stress to the greatest extent possible. When a plant is exposed to high temperatures (thermal stress), the heat transcription factor, also known as the central regulator of the heat shock stress response, regulates the expression of many heat-stress-inducible genes at the transcriptional level by recognizing conserved binding motifs (heat stress element, *HSE*) found in the promoter region, allowing the plant to withstand the stress. *HSPs* also protect cells from heat and other environmental challenges while also contributing to protein folding, which aids protein function, cell differentiation, dimensional structure, and conformation [35]. The tomato (*Lycopersicon esculentum*) is induced by heat shock proteins (*HSPs)* of the leaf induced by the heat treatment and under normal growth conditions of the fruits during the chloroplast transition into chromoplasts [36]. The synthetics of a set of evolutionary polypeptides called heat shock proteins (HSPs) are the response of every organism to high temperatures. Small *HSPs* (*sHSPs*) have a conserved sequence at their C terminus and are connected to other organisms’ HSPs and the crystallins of the vertebrate eye lens. They range in size from 15 to 42 KD [37]. The most abundant and diversified category of proteins generated in response to heat stress is plant *sHSPs*. Other environmental conditions, such as cold, drought, or salinity, can also cause these proteins to be produced, as well as during developmental processes, including embryogenesis, germination, and fruit development [38]. Based on sequence alignments, immunological cross-reactivity, and cellular compartmentalization, *sHSPs* have been divided into six classes. The cytoplasm or nucleus was found in three classes (CI, CII, and CIII). The endoplasmic reticulum, mitochondria, and chloroplast were in other classes. The links between *HSP* synthesis and the stress response led to the belief that these proteins protect cells from the associated high-temperature-damaging consequences [37]. While it has been demonstrated that both plant and human *HSPs* do not have an ATP-dependent chaperone function in vitro, the mechanism by which *sHSPs* are engaged in cell protection is not entirely understood [39]. *sHSPs* acts as chaperones by preventing irreversible aggregation of heat stress denatured proteins in an independent way. As *HSF* genes participate in pathways associated with heat shock, a quantitative PCR analysis has been carried out in using real-time PCR in the tomato for a comprehensive study to detect *HSF* gene expression. The non-conserved regions have been utilized for the first design to ensure the PCR amplification specificity (Table 1). The expression of most *SlyHSF* genes was drastically enhanced with heat stress treatment. After the treatment of heat stress in all samples, the genes, *SlyHSF-01*, *-8*, *-9*, *-10*, and *-11*, have been expressed more under high-temperature conditions while genes *SlyHSF-04*, and *-5* were expressed more under medium or moderate temperature. However, the gene *SlyHSF-02* was expressed more under low-temperature treatment. *SlyHSF-01* and *-02* in tomatoes have been demonstrated to be master regulators for activating heat response and can lead to developing thermotolerance [40]. Similar results were also recorded by Mishra et al., (2002), suggesting that the *HSFA1a* (*SlyHSF-02*) gene represents as the heat response is triggered by a master regulator, which might lead to the development of thermotolerance; however, HSFA1a regulation was not shown to be important in another study [40]. Because HSF genes have been linked to heat shock pathways, quantitative real-time PCR analysis was used to systematically detect HSF gene expression in tomatoes. To ensure the specificity of PCR amplification, non-conserved areas were chosen for the primer design. Moreover, Yang et al. [40] also suggested that different *SlySHFs* genes, such as [02-04-06-16-17-18], had more than ten interaction linkages discovered. However, the *SlyHSF* genes [07-10-12] found that interactions between tomato HSF genes existed. A dynamic protein structure was present in small heat shock protein (sHSPs) having diverse evolutionary origins. The structure is widely used in human diseases, as well as in plants stress acclimatization and other organisms. For that purpose, the ethylene-induced transient structure was used to suppress the HSP genes regulated by the RIN protein, representing their basic role in plant biology, especially in fruit ripening [41,42]. Similar transient suppression results were suggested when the Rd22 gene expression in ABA-attuned plants represents stress tolerance [43]. Another study was conducted to determine the expression and interaction of heat shock proteins in tomato plants in protoplast cells, both with and without heat shock transcription factor A2 (HsfA2) under two different heat stress conditions. The findings show that depending on whether HsfA2, a critical regulator of HSPs, is activated or repressed, distinct sHSPs are upregulated. Furthermore, studies of protein–protein interactions between the sHSP protein family and other heat shock response proteins (HSP70, HSP90, and MBF1c) reveal that numerous sHSPs are mediating alternate stress responses via a regulatory subnetwork independent of HsfA2 [44]. Similarly, HSBP1 was introduced in tomato crops using the TILLING technique to determine the effects of mutation HSBP1 protein functionally at protoplast level and to compare thermotolerance capacity in wild-type tomato genotypes. The results suggested that due to the mutation, the methionine-to-isoleucine mutation partial protein function was lost; as a result, the Hsf inhibitory effect was lost. On the other hand, mutant plants showed thermotolerance; however, matured plants recorded tolerance in HS treatments. Adversely, the wild-type (homozygous) plants or mutant plants recorded no significant differences under the control condition. Cumulatively, the results suggested that identified mutants with HSBP1 can be used as a genetic tool in breeding programs for thermotolerance activity in tomato crops [45]. Under physiological settings, maize EMP2/HSBP1 acts in the early stages of kernel formation, indicating that it has a developmental role [46]. HSBP interacts solely with class A HSFs, and individual HSBPs have distinct specificities for different HsfA members. HsfA2e, HsfA3, HsfA4d, and HsfA5 interact with maize EMP1/HSBP1, whereas HsfA2c and HsfA4a bind to HSBP2 [47]. Transgenic lines reveal changes in the transcript regulation of HSF-dependent genes, such as HSPs, which is consistent with a differential interaction profile that pairs *OsHSBP1* and *OsHSBP2* with different HSFs [48]. Tomatoes are an essential crop for both economic and gastronomic reasons all over the world, and they’ve long been used as a model plant for flesh fruit development [49]. Several HSFs, including HsfA2, were expressed in tomato anthers during the early stages of pollen production, suggesting that SlHSBP1 may play a regulatory function in HSF development [50,51,52]. These findings are consistent with prior research that has revealed that *HSBP1* has a repressor role [53].

## 4. Materials and Methods

### 4.1. Plant Materials

The tomato genotypes used for this study (Table 1) comprised six commercially available cultivars in the local market (Pearson Improved, Strain B, Valentine, Marmande VF, Super Strain B, and Pearson Early) and two local Saudi cultivars (Al-Ahsa and Al-Qatif) from the Plant Gene Bank of the Ministry of Environment, Water and Agriculture (MEWA) in Riyadh, Saudi Arabia. Four-week-old seedlings were transplanted on beds of 100 cm between rows and 50 cm between plants. They were transplanted on three different periods during the 2019 season: period 1 (2 August), period 2 (3 September), and period 3 (1 October). Experiments were conducted at the Research Farm of the Plant Production Department, College of Food and Agriculture Sciences, King Saud University, Riyadh, Saudi Arabia. The soil of the experimental field is characterized as sandy loam with the physicochemical properties summarized in Table 3.

Temperatures were recorded from the on-site weather station. In general, the maximum and minimum mean daily temperatures were 42 °C (summer) and 12 °C (winter), respectively [54]. Growing degree day (*GDD*) equations can transfer climate data into useful agricultural applications that growers can use to make strategic decisions [55]. *GDD*s were calculated during the tomato growing season using the *GDD* model in Equation (1) [56]. The cumulative growing degree days (*CGDD*s), however, were calculated by taking the sum of the *GDD*s as in Equation (2).

(1) $$GDD=\frac{T_{max}+T_{min}}{2}-T_{base}$$

where *T_max_* and *T_min_* are the maximum and minimum daily temperatures (°C), and *T_base_* is the base temperature (10 °C for tomato).

(2) $$CGDD=\overset{n}{\underset{j=1}{\sum }}GDD_{j}$$

where *j* is the indicated day, *n* is a specific day during the growth period, and *GDDj* is the heating unit on a *j*th day (° Cd).

The seeds of the genotypes were sown in JV7 pellets (Jiffy Products, Stange, Norway) under greenhouse environmental conditions at three seeding periods to obtain four weeks of vigorous transplants ready to be transplanted in the field at three scheduled planting dates: 1 August 2019, 1 September 2019, and 1 October 2019. Planting distance was 40 cm between plants and 100 cm between lines (Figure 4). The selection of the planting dates was based on the fact that tomato plants will be exposed to different day/night regimes and therefore, different levels of heat stress during flowering and fruit set. The average temperature, relative humidity, and solar radiation during each growing period are shown in Supplementary Table S1. Climatic data were collected during the agricultural growing seasons implemented in this study, using one of the meteorological stations located within the scope of the study implementation site (Western Nakhil-IRIYADH23). The following were the time frames for each of the planting dates: first planting date (low temperature), 1 August 2019–10 November 2019 (102 days), second planting date (medium temperature), 1 September 2019–25 December 2019 (116 days), and third planting date (high temperature), 1 October 2019–15 February 2019 (138 days).

The maximum and minimum temperatures and the average daily temperature, humidity, and solar radiation were collected during the period from the beginning of July 2019 to the end of February 2020, and then the average weekly temperature was calculated for each month separately. The study used a split-plot in a randomized complete block design with three replications. Season growing treatments were assigned at random to the main plots, while genotypes were assigned to the sub-plots. Three plants from each subplot were randomly selected for the measurements of the vegetative traits in the tomato crop under different temperature regimes.

### 4.2. Measurement of Growth Parameters

The plant’s main stem height was measured from the beginning of the adventitious roots to the top of the plant. The leaf area was measured using the Portable Area Meter (Model LI-3000A, LI-COR, USA). The leaves of the plant were collected and weighed, and then a sample (100 g) was taken and dried at 70 °C in a forced air-oven until the weight became constant (48–72 h), and the dry matter contents were calculated relative to the total fresh weight of the leaves of the plant. The stem of the plant was collected and weighed, and then a sample (100 g) was taken from them and dried at 70 °C in a forced air-oven until the weight became constant (48–72 h), and the dry matter contents were calculated relative to the total fresh weight of the stem of the plant.

### 4.3. Total RNA Extraction

Leaf tissues from the control (30 °C), medium temperature (40 °C), and high temperature (45 °C) plants were collected, immersed in liquid nitrogen, and stored at −80 °C until the RNA was extracted. Using a total RNA extraction kit (SV total RNA isolation system, Promega, Madison, IL, USA), the total RNA was isolated from 100 mg of the control, medium temperature, and high-temperature leaves according to the manufacturer’s recommended technique. Using a mortar and pestle, 100 mg of plant tissue was thoroughly mashed in liquid nitrogen. Tissue powder was transferred to a 2 mL liquid N_2_ cooled microcentrifuge tube that was devoid of RNase. It was then incubated at room temperature for 3 min after adding 450 μL RNA lysis buffer to tissue powder and 350 μL dilution buffer to the lysate. The sample was centrifuged for 10 min at 12,000 rpm following incubation. The clear lysate was then pipetted to a fresh centrifuge tube, 200 μL of 95 percent ethanol was added to clear lysate, and 200 μL of 95 percent ethanol was added to clear lysate and pipetted three to four times. After that, the mixture was transported to a spin column assembly and centrifuged at 12,000 rpm for 2 min. After carefully discarding the flow, 600 μL of RNA washing buffer was added to the spin column, and the membrane was washed by centrifugation for 2 min at 12,000 pm. The flow-through was discarded, and 250 μL of RNA washing buffer was added to wash the RNA before centrifugation at 12,000 rpm for 2 min. The flow-through was discarded, and the samples were treated for 15 min at room temperature with DNase incubation (3 μL) buffer and DNase stop solution. After that, two RNA wash buffer washes were performed (600 μL RNA washing buffer and centrifuged for 2 min). To elute the RNA, 100 μL of RNase-free water was added directly to the middle of the spin column membrane and centrifuged for 1 min at 12,000 rpm. The total RNA quantity was quantified using a Nanodrop 2000 spectrophotometer (Thermo Scientific, Waltham, MA, USA), and the integrity of the RNA was evaluated using a 1.2 percent denaturing formaldehyde agarose gel. RNA was then kept at −80 °C for future use.

### 4.4. cDNA Synthesis

cDNA was synthesized using a superscript III first-strand kit (Thermo Fisher Scientific, Waltham, MA, USA) according to the manufacturer’s instructions. Random hexamer primers and deoxyribonucleotide 5-triphosphate (dNTPs) were added to 5 μg total RNA and the mixture was incubated at 65 °C for 5 min before chilling on ice. The first strand was then reverse transcribed by adding the first strand buffer, 20 mM dithiothreitol, and superscript III reverse transcriptase to a final volume of 20 μL and then incubating the mixture at 42 °C for 1 h. The cDNA obtained was diluted 1:20 (*v*/*v*) with deionized water and stored at −20 °C.

### 4.5. Potential Candidate Genes for Heat Shock Protein in Tomato

The following genes were selected from the literature of Yang et al. [40] from the tomato genome database. The characteristics of the selected genes are presented in Table 1.

### 4.6. Quantitative Real-Time PCR

All selected primers were validated on 10 genotypes based on condition-specific (cold, medium, and heat) stress samples. All the reactions were performed in triplicate on the ABI machine 7500. Actin genes were selected as reference genes due to their consistent expression. Each reaction consisted of 1 μL of cDNA, 10 μL of SYBR green PCR master mix, 0.5 μL each of forward and reversed primers, and water up to 20 μL. The thermocycling protocol consisted of 50 °C for 2 mins, initial denaturation at 95 °C for 10 min, 40 cycles each of denaturation at 95 °C for 0.15 min, and annealing/extension at 56 °C for 30 s. A melting curve analysis protocol was performed to confirm the absence of multiple amplicons and primer dimers. Negative control was also included to ensure the absence of contamination. In addition, the presence of genomic DNA contamination was checked by performing reactions without reverse transcriptase. The PCR efficiency was determined using a standard curve based on five to seven different four-fold dilutions of cDNA cloned amplicon.

### 4.7. Data Analysis

The data were statistically analyzed using the XL-STAT statistical package software (Ver. 2019, Excel Add-ins soft SARL, New York, NY, USA). The cycle’s threshold values were calculated by the software, and data were exported to MS Excel for further analysis. Comparative Ct methods were used to estimate the expression differences. The Ct values of control and stressed samples were normalized with internal standards using the following formula. ∆∆Ct = ∆Ct^target^ (m^Cttarget^-m^Ctendogenous control^) -∆Ct ^control^ (m^Ctcontrol^-m^Ctendogenous control^). The fold difference will be determined using power 2-∆∆Ct. The gene expression data were used to construct the heat map expression (http://www.heatmapper.ca/expression/, accessed on 5 November 2021) profiles [57]; their differential expression was shown in high-temperature stress conditions to validate the study.

## 5. Conclusions

The descriptive statistics results suggested that there was a wide range of differences recorded under three different growing seasons. The qPCR study revealed the expression patterns of HSF genes in tomatoes that followed treatment in high temperate temperatures. Four genes, *SlyHSFs-1*, *SlyHSFs-2*, *SlyHSFs-8*, and *SlyHSFs-9* recorded upregulation expression relative to *SlyHSFs-3*, *SlyHSFs-5*, *SlyHSFs-10*, and *SlyHSFs-11*. The genotypes Strain B, Marmande VF, Pearson’s Early, and the Landrace Al-Qatif-365 recorded the tolerant tomato genotypes under high-temperature stress. These genotypes will be introduced into a breeding program to improve tomato responses to heat stress.

## Figures and Tables

**Table 1:** Descriptive statistics of the studied characteristics of tomato genotypes during three growing seasons.

Traits	Mean	SD	Minimum	Maximum
Plant height (cm)	66.86	6.7	52.90	83
DWL (g)	64.70	8.82	48	85.49
DWS (g)	45.17	6.22	34.26	60.53
FWL(g)	527.28	73.81	396.10	699
FW/p (g)	838.06	117.26	633.73	1114
FWS (g)	310.80	43.43	234.90	415
NoB	6.96	0.86	5.6	8.90
LA/p (cm^2^)	5614.4	778.85	4248.3	7445.8

Plant height (PH), thickness (Th), fresh weight leaf (FWL), fresh weight stem (FWS), number of branches (NOB), fresh weight/plant (FW/p), dry weight leaf (DWL), dry weight stem (DWS), leaf area/plant (LA/p).

**Table 2:** Genomic features of *SlyHSF* Genes in tomato.

ID	Name	Chr #	Start	End	Gene (bp)	# cDNA	# Amino Acid
*SlyHSF-01*	Solyc11g064990.1	SL2.40ch11	47,389,718	47,391,840	2123	756	251
*SlyHSF-02*	Solyc08g005170.2	SL2.40ch08	111,412	116,839	5428	1949	527
*SlyHSF-03*	Solyc03g026020.2	SL2.40ch03	7,810,489	7,812,280	1792	1594	338
*SlyHSF-04*	Solyc03g097120.2	SL2.40ch03	52,901,766	52,904,929	3164	1874	491
*SlyHSF-05*	Solyc02g090820.2	SL2.40ch02	46,880,125	46,883,382	3258	1611	301
*SlyHSF-06*	Solyc09g065660.2	SL2.40ch09	59,473,864	59,475,995	2132	1405	372
*SlyHSF-07*	Solyc04g078770.2	SL2.40ch04	61,036,586	61,037,903	1318	1230	360
*SlyHSF-08*	Solyc06g072750.2	SL2.40ch06	41,255,352	41,258,348	2997	1613	482
*SlyHSF-09*	Solyc12g098520.1	SL2.40ch12	48,549,454	48,552,229	2776	1437	478
*SlyHSF-10*	Solyc08g080540.2	SL2.40ch08	60,985,869	60,987,278	1410	1329	325
*SlyHSF-11*	Solyc04g016000.2	SL2.40ch04	6,594,909	6,598,451	3543	1320	237

**Table 3:** Physical characteristics of soil.

Soil Texture				pH	EC dS m^−1^	Anions (mEq L^−1^)				Cations (mEq L^−1)^		
Clay (%)	Silt (%)	Sand (%)	Soil Type			Ca	Mg	K	Na	HCO_3_	Cl	SO_4_
8.45	7.83	83.72	Sandy Loam	7.8	1.98	10.50	4.50	1.32	6.97	2.30	2.65	18.34

## Data Availability

All the data supporting this article were included in the main text.

## Supplementary Materials

The following are available online at https://www.mdpi.com/article/10.3390/plants11050615/s1, Table S1: Mean data for all genotypes under three growing seasons.
